# Supplementary material for: Changes in self-reported health and wellbeing outcomes in 36,951 primary school children from 2014 to 2022 in Wales: an analysis using annual survey data
Source: Front Public Health. 2024 Feb 14;12:1285687. doi: 10.3389/fpubh.2024.1285687 (PMC10899516; doi:10.3389/fpubh.2024.1285687)
Supplement: Supplementary file 1 [file Data_Sheet_1.DOCX]

Supplementary File 3

| Variable | Purpose | Coding |
| --- | --- | --- |
| GENDER | Demographic | 1 = Boy  2 = Girl  9 = Prefer Not To Say |
| FruitVeg | Diet | How many portions of fruit and veg did you eat yesterday? |
| PA60min | Physical activity | How many days were you active for 60 mins?  0 = 0 days  1 = 1-2 days  2 = 3-4 days  3 = 5-6 days  4 = 7 days |
| Tired | Sleep | How many days did you feel tired?  0 = 0 days  1 = 1-2 days  2 = 3-4 days  3 = 5-6 days  4 = 7 days |
| SugarySnacks | Diet | How many days did you have sugary snacks?  0 = 0 days  1 = 1-2 days  2 = 3-4 days  3 = 5-6 days  4 = 7 days |
| RideaBike | Physical activity | Can you ride a bike without stabilisers?  0 = No  1 = Yes |
| Swim25m | Physical activity | Can you swim 25m without armbands?  0 = No  1 = Yes |
| ChildWBFriends | Wellbeing | On a scale of 1 – 10 how happy are you with: Your Friends |
| GeneralCompetency | Wellbeing | There are lots of things I am good at (Strongly agree, agree etc.)  1 = Strongly disagree  2 = Disagree  3 = Agree  4 = Strongly agree |
| Me and My Feelings Questionnaire (Deighton et al. 2012):  Mhfeellonely (I feel lonely)  Mhcrylot (I cry a lot)  Mhunhappy (I am unhappy)  Mhnobodylikesme (I feel nobody likes me)  Mhworrylot (I worry a lot)  Mhproblemsleeping (I have problems sleeping)  Mhwakeupinnight (I wake up in the night)  Mhshy (I am shy)  Mhfeelscared (I feel scared)  Mhworryatschool (I worry when I am at school)  Mhgetveryangry (I get very angry)  Mhlosetemper (I lose my temper)  Mhhitout (I hit out when I am angry)  Mhdothingstohurt (I do things to hurt people)  Mhcalm (I am calm)  Mhbreakthings (I break things on purpose)  (Measures emotional/behavioural difficulties) | Wellbeing | 0 = Never  1 = Sometimes  2 = Never |
| emotionaldifficulty | Wellbeing | Continuous score for emotional difficulty |
| behaviouraldifficulty | Wellbeing | Continuous score for behavioural difficulty |
| safeplayinginarea | Environment | On a scale of 1 – 10 how safe do you feel in your area |
| physlitgood | Physical activity | I am good at lots of activities (Strongly agree, agree etc.)  1 = Strongly disagree  2 = Disagree  3 = Agree  4 = Strongly agree |
| physlitunderstand | Physical activity | I understand why activity is good for me (Strongly agree, agree etc.)  1 = Strongly disagree  2 = Disagree  3 = Agree  4 = Strongly agree |
| WIMD2019Quartile | Environment | WIMD Quartile |
| HaveFriendsOver | Environment | Did someone else come to your house in the last week?  0 = 0 days  1 = 1-2 days  2 = 3-4 days  3 = 5-6 days  4 = 7 days |
