# Supplementary material for: Changes in self-reported health and wellbeing outcomes in 36,951 primary school children from 2014 to 2022 in Wales: an analysis using annual survey data
Source: Front Public Health. 2024 Feb 14;12:1285687. doi: 10.3389/fpubh.2024.1285687 (PMC10899516; doi:10.3389/fpubh.2024.1285687)
Supplement: Supplementary file 3 [file Data_Sheet_3.DOCX]

THE HAPPEN SURVEY

[AT HOME]

Consent Form

Before you start please click this link to read the information sheet...

<https://happen-wales.co.uk/childrens-information-sheet/>

1. I have read the child information sheet and understand that if I take part I can change my mind at any time, and this will not be a problem at all. *

*Mark only one oval.*

- Yes
- No

2. I am happy for you to use my questionnaire for research. Only the researchers in the team will know my name and will not tell anyone else my answers *

*Mark only one oval.*

- Yes
- No do not use my questionnaire

3. I am happy for you to look at my school and health records to see how my school is doing (as a group). This is anonymous which means I cannot be identified *

*Mark only one oval.*

- Yes
- No

If you do not wish to take part in the questionnaire please do not continue.

Please click next to start the questionnaire!

ABOUT YOU

4. First Name*

5. Last Name*

6. Home Post Code*

7. What school do you go to?*

8. Are you still going to your school?*

*Mark only one oval.*

- No, I am at home
- Yes, most days of the week
- Yes, sometimes
- I am in a different school from my own school

9. Do you have any other children living in your house with you (brothers, sisters) ?

*Mark only one oval.*

- Yes
- No

10. How many people live in your home with you (including adults)?

- 1
- 2
- 3
- 4
- 5
- 6+

11. What year are you in now?*

*Mark only one oval.*

- Year 4
- Year 5
- Year 6

12. Gender*

*Mark only one oval.*

- Boy
- Girl
- Prefer not to say

13. Date of Birth

Year*

*Mark only one oval.*

- 2007
- 2008
- 2009
- 2010
- 2011
- 2012

14. Month*

*Mark only one oval.*

- January
- February
- March
- April
- May
- June
- July
- August
- September
- October
- November
- December

15. Day *

*Mark only one oval.*

- 1
- 2
- 3
- 4
- 5
- 6
- 7
- 8
- 9
- 10
- 11
- 12
- 13
- 14
- 15
- 16
- 17
- 18
- 19
- 20
- 21
- 22
- 23
- 24
- 25
- 26
- 27
- 28
- 29
- 30
- 31

YESTERDAY

16. What did you eat for breakfast YESTERDAY?

*Check all that apply.*

- Nothing
- Cereal like cocopops, frosties, sugar puffs, chocolate cereals
- Healthy cereal like e.g. porridge, weetabix, readybrek, muesli, branflakes, cornflakes
- Snacks like biscuits
- Fruit
- Toast
- Cooked breakfast
- Yoghurt
- Other:

17. Did you eat any fruit and veg YESTERDAY?

*Mark only one oval.*

- No
- 1 piece
- 2 or more fruit and veg

18. How many times did you brush your teeth YESTERDAY?

*Mark only one oval.*

- 0
- 1
- 2
- 3

19. What time did you fall asleep YESTERDAY (to the nearest half hour)?

*Mark only one oval.*

- 6.00pm
- 6.30pm
- 7:00pm
- 7:30pm
- 8:00pm
- 8:30pm
- 9:00pm
- 9:30pm
- 10:00pm
- 10:30pm
- 11:00pm
- 11:30pm
- 12:00am
- 12:30am
- 1:00am
- 1:30am
- 2:00am
- 3:00am
- 3:30am
- 4:00am

20. What time did you wake up TODAY (to the nearest half hour)?

*Mark only one oval.*

- 5:00am
- 5:30am
- 6:00am
- 6:30am
- 7:00am
- 7:30am
- 8:00am
- 8:30am
- 9:00am
- 9.30am
- 10.00am
- 10.30am
- 11.00am
- 11.30am

THE LAST WEEK

NOW think about what you did in the last 7 days...

21. In the last 7 days, how many days did you do sports or exercise for at least 1 hour in total. This includes doing any activities (including online activities like Joe Wicks) or playing sports where your heart beat faster, you breathed faster, and you felt warmer?

*Mark only one oval.*

- 0 days
- 1-2 days
- 3-4 days
- 5-6 days
- 7 days

22. In the last 7 days, how many days did you watch TV/play online games/use the internet etc. for 2 or more hours a day (in total)?

*Mark only one oval.*

- 0 days
- 1-2 day
- 3-4 days
- 5-6 days
- 7 days

23. In the last 7 days, how many days did you feel tired?

*Mark only one oval.*

- 0 days
- 1-2 days
- 3-4 days
- 5-6 days
- 7 days

24. In the last 7 days, how many days did you feel like you could concentrate/pay attention well on your schoolwork?

*Mark only one oval.*

- 0 days
- 1-2 days
- 3-4 days
- 5-6 days
- 7 days
- Don’t do school work

25.  In the last 7 days, how many days did you drink at least one fizzy drink (e.g. coke, fanta, sprite)
 *Mark only one oval.*

- 0 days
- 1-2 days
- 3-4 days
- 5-6 days
- 7 days

26. In the last 7 days, how many days did you eat at least one sugary snack (e.g. chocolate bar, sweets)
 *Mark only one oval.*

- 0 days
- 1-2 days
- 3-4 days
- 5-6 days
- 7 days

27. In the last 7 days, how many days did you eat take away foods (e.g. Chinese takeaway)

*Mark only one oval.*

- 0 days
- 1-2 days
- 3-4 days
- 5-6 days
- 7 days

ACTIVITY AND YOUR AREA

28. On a scale of 0 to 10 (0 being not very safe and 10 being very safe), how safe do you feel playing in your area?

*Mark only one oval.*

- 0
- 1
- 2
- 3
- 4
- 5
- 6
- 7
- 8
- 9
- 10

29. From your house, can you easily walk to a park (for example a field, grassy area)?

*Mark only one oval.*

- Yes
- No

30. From your house, can you easily walk to somewhere you can play?
 *Mark only one oval.*

- Yes
- No

31. Do you have a garden?

- Yes
- No

32. How often do you go out to play outside?

*Mark only one oval.*

- Most days
- A few days each week
- Hardly ever
- I don’t play

33. Do you have enough time for play?

*Mark only one oval.*

- Yes, I have loads
- Yes, it’s just about enough
- No, I would like to have a bit more
- No, I need a lot more

34. What type of places do you play in?

- In my house
- In my garden
- In the street
- On a local grassy area
- In a place with bushes, trees and flowers
- In the woods near my house
- On a football field near my house
- In my school playground
- Somewhere with water or sand in it
- On the bike or skate park
- Somewhere else:

35. Can you play in all the places you would like to?

- I can play in all the places I would like to
- I can play in some of the places I would like to
- I can only play in a few places I would like to
- I can hardly play in any of the places I would like to

36. Do you have somewhere at home where you have space to relax

Yes

Sometimes but not all the time

No

YOU AND YOUR FEELINGS

This part of the survey is going to ask you how you feel. There are no right or wrong answers. You should just pick the answer which is best for you.

37. Tell us if you agree or disagree with the following:

*Mark only one oval per row.*

I am doing well with my schoolwork

- Strongly agree
- Agree
- Don't agree or disagree
- Disagree
- Strongly disagree
- I don’t know
- I feel part of my school community Strongly agree
- Agree
- Don't agree or disagree
- Disagree
- Strongly disagree
- I don’t know

I have lots of choice over things that are important to me

- Strongly agree
- Agree
- Don't agree or disagree
- Disagree
- Strongly disagree

There are lots of things I'm good at

- Strongly agree
- Agree
- Don't agree or disagree
- Disagree
- Strongly disagree

38. On a scale of 0 to 10 (0 being very unhappy and 10 being very happy), how do you feel about

*Based on the Good Childhood Index by the Children's Society

39. Your Health

*Mark only one oval.*

- 0
- 1
- 2
- 3
- 4
- 5
- 6
- 7
- 8
- 9
- 10

40. Your Family

*Mark only one oval.*

- 0
- 1
- 2
- 3
- 4
- 5
- 6
- 7
- 8
- 9
- 10

41. Your Friends

*Mark only one oval.*

- 0
- 1
- 2
- 3
- 4
- 5
- 6
- 7
- 8
- 9
- 10

42. Your Appearance (how you look)

*Mark only one oval.*

- 0
- 1
- 2
- 3
- 4
- 5
- 6
- 7
- 8
- 9
- 10

43. Your Life

*Mark only one oval.*

- 0
- 1
- 2
- 3
- 4
- 5
- 6
- 7
- 8
- 9
- 10

ME AND MY FEELINGS

Based on the Me and My Feelings Questionnaire ( Deighton, Tymms, Vostanis, Belsky, Fonagy, Brown, Martin, Patalay, & Wolpert, 2012)

44. Remember, there are no right or wrong answers, just pick which is right for you.

*Mark only one oval per row.*

I feel lonely

- Never
- Sometimes
- Always

I cry a lot

- Never
- Sometimes
- Always

I am unhappy

- Never
- Sometimes
- Always

I feel nobody likes me

- Never
- Sometimes
- Always

I worry a lot

- Never
- Sometimes
- Always

I have problems sleeping

- Never
- Sometimes
- Always

I wake up in the night

- Never
- Sometimes
- Always

I am shy

- Never
- Sometimes
- Always

I feel scared

- Never
- Sometimes
- Always

I worry when I am at school

- Never
- Sometimes
- Always

I get very angry

- Never
- Sometimes
- Always

I lose my temper

- Never
- Sometimes
- Always

I hit out when I am angry

- Never
- Sometimes
- Always

I do things to hurt people

- Never
- Sometimes
- Always

I am calm

- Never
- Sometimes
- Always

I break things on purpose

- Never
- Sometimes
- Always

45. Are you able to keep in touch with your family that you don’t live with?

*Mark only one oval.*

- Yes
- No

46. Are you able to keep in touch with your friends?

*Mark only one oval.*

- Yes
- No

47. If yes, how are you keeping in touch (tick all that are relevant)?

- Live near them so I can see them (at a social distance)
- By phone (texting, calling or video calling)
- On social media
- On games consoles

SUBMIT

Don't forget to press submit below! Once you've pressed submit you are all done!

If you'd like some additional resources while you're at home during COVID-19, we've put together some here:

<https://happen-wales.co.uk/some-resources-for-you/>
